# How Do We Manage HLA-B27-associated Ocular Inflammation Refractory or Intolerant to Conventional Immunomodulatory Therapy?

**DOI:** 10.18502/jovr.v15i4.7777

**Published:** 2020-10-25

**Authors:** Doan Luong Hien, Brandon Huy Pham, Quan Dong Nguyen

**Affiliations:** ^1^Spencer Center for Vision Research, Byers Eye Institute, Stanford University, Palo Alto, California, USA; ^2^Pham Ngoc Thach University of Medicine, Saigon, Vietnam

The HLA-B27 gene is among the most studied genes in the history of medicine, and its relationship to ocular inflammation is well established. In particular, it has been known to be associated primarily with anterior chamber inflammation with clinical manifestations of non-granulomatous keratic precipitates, anterior chamber cells and flare, and in some cases, fibrin and/or hypopyon. With modern advanced imaging technologies, posterior segment involvement, including papillitis and retinal vasculitis can be detected in up to 31% of patients with HLA-B27-associated uveitis.^[1]^ Moreover, wide-angle imaging has allowed the diagnosis of peripheral retinal vasculitis that may be missed by standard imaging modalities.^[2,3]^

Although the long-term visual prognosis of HLA-B27-associated acute anterior uveitis (AAU) is generally favorable,^[4]^ patients with HLA-B27-associated AAU are approximately five times more likely to have a visual acuity of 20/200 or worse as compared to patients without HLA-B27-positivity.^[5]^ Suboptimal visual outcomes may be complicated by steroid-induced side effects or delay in treatment of refractory cases; therefore, close monitoring with multimodality imaging and employing a stepladder approach in the management is necessary in every patient. Unfortunately, since relatively few studies have examined HLA-B27-associated AAU, and even fewer have focused on refractory cases, HLA-B27-associated AAU remains a significant therapeutic challenge for uveitis specialists. In their well-written manuscript and well-designed study published in the current issue of *Journal of Ophthalmic and Vision Research (JOVR)*, Bajwa and colleagues^[6]^ contribute to the literature by discussing the utility of infliximab in managing this particularly challenging disease.

Recent prospective randomized controlled trials have shown that intraocular inflammation can be controlled in 57.1–66.7% of cases with first-line immunomodulatory therapy (IMT) agents, such as methotrexate and mycophenolate mofetil.^[7]^ Uveitis that involves the posterior segment may not always respond to first-line IMT and at time requires adjustment to second- or third-line agents, including biologics or other steroid-sparing agents. Infliximab and adalimumab are the two most commonly used biologic agents for noninfectious posterior uveitis (NIU). Unlike adalimumab, infliximab has not been approved by the FDA for NIU and is used off-label for ocular inflammation. Data supporting the use of infliximab in NIU stems largely from retrospective and small prospective trials.^[8,9,10,11,12,13,14,15,16,17]^ Infliximab can be used as first-line therapy for certain systemic diseases such as Adamantiades-Behçet disease^[18]^ and in cases of sight-threatening disease in the setting of moderate to severe idiopathic retinal vasculitis and optic disc inflammation, or as a third-line therapy in uveitis refractory to corticosteroids and conventional IMT. The efficacy of infliximab is fairly rapid-onset, with one study demonstrating 96% resolution of acute inflammation one day after infusion,^[19]^ which is quite fast as compared to adalimumab, in which the typical time to effectiveness typically ranges from 2 to 16 weeks.^[20]^ These findings are consistent with the study by Bajwa et al,^[6]^ which demonstrated 81.25% responsiveness after three months and 87.5% responsiveness after six months.

Some IMT agents, such as methotrexate and mycophenolate mofetil, may take 8–12 weeks before reaching maximum efficacy; adalimumab takes a median of six weeks.^[20]^ Therefore, three months of follow-up is inadequate to determine unresponsiveness. We typically follow our patient in the clinics for six months or more to fully assess drug efficacy and responsiveness. It is important to continue therapy during this time even if the disease is in stable condition in order to achieve long-term quiescence and remission. Adalimumab was FDA-approved for the treatment of NIU after the completion of two successful phase-3 multicenter randomized controlled trials, VISUAL I and II, that investigated the use of adalimumab strictly for intermediate, posterior, or pan-uveitis.^[21,22]^ No specific data regarding the percentage and subtypes of HLA-B27-associated uveitis is available from the VISUAL I and II studies. It is quite interesting that many patients had refractory anterior uveitis in the Bajwa study^[6]^ prior to the study entry and 20.8% were considered to be unresponsive or intolerant to adalimumab therapy. It would be beneficial if Bajwa and colleagues could provide information on prior immunosuppression treatment regimens including route, time, and dosage. Moreover, no clear definition of “unresponsive inflammation” is provided. According to the study, 9.5–19% of patients experienced a flare up while on treatment with infliximab, and one patient developed vasculitis after 3 months of treatment which remained active until 24 months. We suspect that the authors may be more in favor of infliximab than other IMT agents, having kept the patient on a similar treatment regimen.

In addition, the authors discuss antibody formation against infliximab. It would be very helpful to know whether testing for this antibody was performed as well as how many patients were on concurrent IMT (such as methotrexate or mycophenolate) to prevent or decrease the risk of antibody formation. In the Bajwa study,^[6]^ treatment was prematurely stopped in three patients, and one patient still had active disease at the end of 24 months. The authors can speculate or suggest what may be the next treatment option(s) for these patients.

In summary, while infliximab is a robust treatment, roughly 10–20% of patients may not show an adequate response to therapy. These patients may need augmentation with additional therapeutic approaches. Recent emerging and adopted therapies, including tocilizumab (STOP study),^[23]^ sarilumab (SATURN study),^[24]^ and sirolimus (SAVE-2 and SAKURA studies),^[25,26,27,28]^ have shown encouraging efficacy outcomes with a relatively favorable safety profile. Other clinical trials evaluating the safety and efficacy of filgotinib,^[29]^ tofacitinib,^[30]^ and adrenocorticotropic hormone^[31]^ in NIU are currently in progress.

Amidst the current global COVID-19 pandemic, one of the most common concerns we have received from patients on IMT is whether their treatment might increase the risk of worsening a COVID-19 infection if they were to contract severe acute respiratory syndrome coronavirus 2 (SARS-CoV-2). Currently, there is no clear evidence to suggest that IMT for ocular diseases increases the risk for infection or complications from COVID-19. Although further studies are needed, perhaps tocilizumab, which has recently been shown to reduce risk of death in patients with severe COVID-19 disease,^[32,33]^ can be considered as an alternative treatment option for patients with NIU who fail therapy with infliximab.

##  Financial Support and Sponsorship

DLH and BHP have no relevant funding disclosures. QDN and his employer, Stanford University, have received research funding from Genentech, Gilead, Regeneron, and Santen, among others.
